# Diagnosis, Prevalence, Awareness, Treatment, Prevention, and Control of Hypertension in Cameroon: Protocol for a Systematic Review and Meta-Analysis of Clinic-Based and Community-Based Studies

**DOI:** 10.2196/resprot.7807

**Published:** 2017-05-29

**Authors:** Barthelemy Kuate Defo, Jean Claude Mbanya, Jean-Claude Tardif, Olugbemiga Ekundayo, Sylvie Perreault, Louise Potvin, Robert Cote, Andre Pascal Kengne, Simeon Pierre Choukem, Felix Assah, Samuel Kingue, Lucie Richard, Roland Pongou, Katherine Frohlich, Jude Saji, Pierre Fournier, Eugene Sobngwi, Valery Ridde, Marie-Pierre Dubé, Simon De Denus, Wilfred Mbacham, Jean-Philippe Lafrance, Dickson Shey Nsagha, Warner Mampuya, Anastase Dzudie, Lyne Cloutier, Christina Zarowsky, Agatha Tanya, Paul Ndom, Marie Hatem, Evelyne Rey, Louise Roy, Roxane Borgès Da Silva, Christian Dagenais, David Todem, Robert Weladji, Dora Mbanya, Elham Emami, Zakariaou Njoumemi, Laurence Monnais, Carl-Ardy Dubois

**Affiliations:** ^1^ Department of Social and Preventive Medicine Department of Demography and Public Health Research Institute Université de Montréal Montreal, QC Canada; ^2^ Department of Internal Medicine and Specialities Faculty of Medicine and Biomedical Sciences University of Yaounde I Yaounde Cameroon; ^3^ Montreal Heart Institute Université de Montréal Montreal, QC Canada; ^4^ Department of Public Health and Health Administration College of Health Science and Public Health Eastern Washington University Spokane, WA United States; ^5^ Faculté de Pharmacie Université de Montréal Montreal, QC Canada; ^6^ Department of Social and Preventive Medicine Université de Montréal Montreal, QC Canada; ^7^ Departments of Neurology, Neurosurgery and Medicine McGill University Montreal, QC Canada; ^8^ Department of Medicine University of Cape Town Cape Town South Africa; ^9^ Department of Internal Medicine and Paediatrics Faculty of Health Sciences University of Buea Buea Cameroon; ^10^ Department of Public Health Faculty of Medicine and Biomedical Sciences University of Yaounde I Yaounde Cameroon; ^11^ Department of Cardiology Faculty of Medicine and Biomedical Sciences University of Yaounde I Yaounde Cameroon; ^12^ Faculté des sciences infirmières Université de Montréal Montreal, QC Canada; ^13^ Department of Economics University of Ottawa Ottawa, ON Canada; ^14^ Public Health Research Institute School of Public Health Université de Montréal Montreal, QC Canada; ^15^ Department of Biochemistry and Physiology Faculty of Medicine and Biomedical Sciences University of Yaounde I Yaounde Cameroon; ^16^ Faculté de médecine et Faculté de pharmacologie Université de Montréal Montreal, QC Canada; ^17^ Department of Public Health Hygiene Faculty of Health Sciences University of Buea Buea Cameroon; ^18^ Faculté de Médecine Université de Sherbrooke Sherbrooke, QC Canada; ^19^ Faculty of Health Sciences University of Buea Buea Cameroon; ^20^ Département des sciences infirmières Université du Québec à Trois-Rivières Trois-Rivières, QC Canada; ^21^ College of Technology University of Bamenda Bamenda Cameroon; ^22^ Department of Oncology Faculty of Medicine and Biomedical Sciences University of Yaounde I Yaounde Cameroon; ^23^ Faculty of Medicine and CHU Sainte-Justine Université de Montréal Montreal, QC Canada; ^24^ Service de néphrologie (CHUM-Saint-Luc) & Faculté de médecine Université de Montréal Montreal, QC Canada; ^25^ Département de psychologie Université de Montréal Montreal, QC Canada; ^26^ Department of Epidemiology and Biostatistics Michigan State University East Lansing, MI United States; ^27^ Department of Biology Concordia University Montreal, QC Canada; ^28^ Department of Haematology Faculty of Medicine and Biomedical Sciences University of Yaounde I Yaounde Cameroon; ^29^ Faculty of Dental Medicine Université de Montréal Montreal, QC Canada; ^30^ Département d'histoire Université de Montréal Montreal, QC Canada

**Keywords:** hypertension, prevention, control, diagnosis, awareness, treatment, medication adherence, management, comorbidity, ecology, Cameroon, Africa, systematic review, meta-analysis, protocol

## Abstract

**Background:**

Hypertension holds a unique place in population health and health care because it is the leading cause of cardiovascular disease and the most common noncommunicable condition seen in primary care worldwide. Without effective prevention and control, raised blood pressure significantly increases the risk of stroke, myocardial infarction, chronic kidney disease, heart failure, dementia, renal failure, and blindness. There is an urgent need for stakeholders—including individuals and families—across the health system, researchers, and decision makers to work collaboratively for improving prevention, screening and detection, diagnosis and evaluation, awareness, treatment and medication adherence, management, and control for people with or at high risk for hypertension. Meeting this need will help reduce the burden of hypertension-related disease, prevent complications, and reduce the need for hospitalization, costly interventions, and premature deaths.

**Objective:**

This review aims to synthesize evidence on the epidemiological landscape and control of hypertension in Cameroon, and to identify elements that could potentially inform interventions to combat hypertension in this setting and elsewhere in sub-Saharan Africa.

**Methods:**

The full search process will involve several steps, including selecting relevant databases, keywords, and Medical Subject Headings (MeSH); searching for relevant studies from the selected databases; searching OpenGrey and the Grey Literature Report for gray literature; hand searching in Google Scholar; and soliciting missed publications (if any) from relevant authors. We will select qualitative, quantitative, or mixed-methods studies with data on the epidemiology and control of hypertension in Cameroon. We will include published literature in French or English from electronic databases up to December 31, 2016, and involving adults aged 18 years or older. Both facility and population-based studies on hypertension will be included. Two reviewers of the team will independently search, screen, extract data, and assess the quality of selected studies using suitable tools. Selected studies will be analyzed by narrative synthesis, meta-analysis, or both, depending on the nature of the data retrieved in line with the review objectives.

**Results:**

This review is part of an ongoing research program on disease prevention and control in the context of the dual burden of communicable and noncommunicable diseases in Africa. The first results are expected in 2017.

**Conclusions:**

This review will provide a comprehensive assessment of the burden of hypertension and control measures that have been designed and implemented in Cameroon. Findings will form the knowledge base relevant to stakeholders across the health system and researchers who are involved in hypertension prevention and control in the community and clinic settings in Cameroon, as a yardstick for similar African countries.

**Trial Registration:**

PROSPERO registration number: CRD42017054950; http://www.crd.york.ac.uk/PROSPERO/ display_record.asp?ID=CRD42017054950 (Archived by WebCite at http://www.webcitation.org/6qYSjt9Jc)

## Introduction

### Rationale

The term “blood pressure” (BP) was coined over 300 years ago by the British Reverend Stephen Hales, who first measured it [1]; yet our understanding of the pathogenesis, consequences, and treatments of hypertension (or raised BP, or elevated BP, or high BP) had remained greatly limited and inadequate until only the past four decades or so [2]. Moreover, despite improvements in the prevention of hypertension and control of BP in patients with hypertension, the condition remains a “silent killer” of major public health significance globally [3-6], because these improvements have not always been tangibly extended from the individual patient to the entire population [7,8].

As one of the most important preventable contributors to the global burden of disease and death [9], high BP remains a major risk factor for cardiovascular and renal morbidity and mortality [10-12], and systolic blood pressure (SBP) is associated with the highest burden among cardiovascular risk factors above and beyond either smoking or obesity [13]. Hypertension leads to myocardial infarction, stroke, renal failure, and death if not detected early and treated appropriately [14-18]. There is also a close relationship between BP levels and the risk of cardiovascular events, strokes, and kidney disease: the risk of these outcomes is lowest at a BP of around 115/75 mm Hg, whereas above 115/75 mm Hg, for each increase of 20 mm Hg in SBP or 10 mm Hg in diastolic blood pressure (DBP), the risk of major cardiovascular and stroke events doubles [19]. In 2010, hypertension was responsible for 9.4 million deaths and 7% of the global disease burden as measured in disability-adjusted life-years [20]. Between 1990 and 2015, the global estimated annual death rate per 100,000 associated with high SBP increased from 97.9 (95% uncertainty interval [UI] 87.5-108.1) to 106.3 (95% UI 94.6-118.1), and the loss of disability-adjusted life-years associated with high SBP increased from 5.2 million (95% UI 4.6-5.7 million) to 7.8 million (95% UI 7.0-8.7 million) [21]. During the same period, hypertension was responsible for the largest numbers of deaths worldwide caused by ischemic heart disease (4.9 million, 95% UI 4.0-5.7 million; 54.5%), hemorrhagic stroke (2.0 million, 95% UI 1.6-2.3 million; 58.3%), and ischemic stroke (1.5 million, 95% UI 1.2-1.8 million; 50.0%) [21].

There is an urgent need for stakeholders—including patients and families—across the health system, researchers, and decision makers to work collaboratively to improve prevention, screening and detection, diagnosis and evaluation, awareness, treatment and medication adherence, management, and control for people with or at high risk for hypertension, thereby preventing complications and reducing the need for hospitalization, costly interventions such as cardiac bypass surgery and dialysis, and premature deaths. In rapidly changing socioeconomic, demographic, epidemiological, nutritional, cultural, and physical environments typical of many resource-constrained countries in sub-Saharan Africa (SSA) such as Cameroon, the evidence on the epidemiology and control of noncommunicable diseases (NCDs) in general and hypertension in particular is scattered and poorly documented [4,8,22-25]. High BP contributes to 75% of all strokes and heart attacks [26]; in Cameroon, stroke (4.6%) and ischemic heart disease (3.8%) are the top killers among all NCDs and the fifth and sixth cause of death, respectively, preceded by human immunodeficiency virus (HIV)/AIDS (13.4%), lower respiratory infections (12.2%), diarrheal diseases (6.0%) and malaria (5.0%) [27]. In such environments, the distribution of hypertension-related disease and its population impact are expected to be geographically heterogeneous with variations in ecological setting, population aging, rate of urbanization, and unhealthy lifestyles [11,12,28-32].

We plan to identify and systematically synthesize available data on hypertension in Cameroon in order to generate new knowledge and produce up-to-date information designed to (1) provide a countrywide portrait on estimates and influential factors of prevention, screening and detection, diagnosis and evaluation, awareness, treatment and medication adherence, management, and BP therapeutic (pharmacological and nonpharmacological) interventions for people with or at high risk for hypertension; (2) assess variation in hypertension prevention and control at both the population and health care organization levels across regions and ecologies within Cameroon; and (3) identify data needs, research gaps, user and patient needs, and needs of primary care clinicians for BP treatment for people with hypertension wherever they are located, to support national hypertension prevention and control efforts that can improve patient outcomes.

Thus, this review seeks to appraise evidence in order to provide valuable information that will contribute to facilitating the design, implementation, or furthering of strategies for the epidemiology and control of hypertension in various settings and across at-risk population subgroups in Cameroon, given the changing scope of public health within constantly altering global, national, and local health ecologies.

### Objectives

We will undertake a systematic review of the epidemiology and control of hypertension in Cameroon using a broad set of eligibility criteria and a thorough search strategy. We aim to (1) review overall and within-country diagnostic practices, prevalence, awareness, treatment, prevention, and control of hypertension; and (2) examine the pattern and disparities of this condition across different socioeconomic and demographic groups, and areal and agroecological milieus. Clinical practice guidelines in resource-limited settings should be designed, developed, and improved within the context of areal and sociocultural realities of the health care system and its stakeholders, including the individual patient, so as to embody and support the interrelationships among enduring behavioral and structural changes in societies that are critical contributors to hypertension risk and clinical decision making. Rather than dictating a one-size-fits-all approach to hypertension prevention and patient care, nationally appropriate clinical practice guidelines are most suitable to enhance culturally appropriate prevention strategies and measures targeted at the general population, as well as clinician and patient decision making, by clearly describing and appraising the evidence and the likely benefits and harms behind recommendations for hypertension prevention in adulthood and control of BP in people with hypertension.

Because health occurs within constantly changing socioeconomic and health care environments in much of SSA, this review will address two high-stake questions with broad population health and health care impacts in resource-limited settings such as Cameroon: (1) What is the epidemiological landscape of hypertension among adults aged 18 years or older in Cameroon? (2) What control strategies have been designed and implemented in clinic or community settings to reduce the burden of hypertension in a socioculturally and environmentally diverse setting such as Cameroon?

## Methods

### Protocol and Registration

Many guidelines in health and health care are based on systematic review of the evidence. Before undertaking this systematic review, we searched the Database of Abstracts of Reviews of Effects, the Cochrane Database of Systematic Reviews, the National Institute for Health and Care Excellence website, the National Institute for Health Research Health Technology Assessment program website, the Campbell Collaboration website, the Evidence for Policy and Practice Information and Co-ordinating-Centre (a database of systematic and nonsystematic reviews of public health interventions, or database of promoting health effectiveness reviews), the National Guideline Clearinghouse, the previous year (2015) of Medline, and other appropriate bibliographic databases that could be helpful in identifying recently published reviews. We found no existing or ongoing reviews.

The planned review is being spearheaded by members of our International and Interdisciplinary Research Team on the Dual Burden of Communicable and Noncommunicable Diseases in Africa forming the review team. They have expertise in systematic reviews and meta-analysis, hypertension, primary care (including geriatrics, cardiology, nephrology, nursing, and pharmacology), research (including clinical trials), evidence-based medicine, preventive medicine, epidemiology, informatics and biostatistics, development and implementation of clinical guidelines in systems of care, and other important fields relevant to hypertension prevention and control in high-income countries (HICs), as well as low- and middle-income countries (LMICs).

This protocol conforms to the Preferred Reporting Items for Systematic Review and Meta-Analysis Protocols guidelines [33]. Our systematic review rationale and methods were specified in advance and documented in the PROSPERO register on January 16, 2017 (CRD42017054950).

### Eligibility Criteria

We will select studies according to the two general categories of eligibility criteria (ie, study characteristics and report characteristics) outlined below for the planned review. This review will seek information on the epidemiological landscape of hypertension in terms of geographical coverage (ie, rural, urban, or both), agroecological zones and region of residence of study participants, and costs (at the individual, family, community, and national levels) on the one hand, and the control of hypertension on the other hand.

#### Population

We will include studies examining the general adult Cameroonian population, or healthy adult Cameroonians, or those with hypertension and related comorbidities aged 18 years or older, who consented to participate in studies that will be reviewed. To be selected, a quantitative study must include a group of 10 or more participants, since at least 10 data points per predictor parameter are required in a regression model. We will exclude unpublished articles, commentaries, editorials, newspaper articles, theses, conference abstracts, and conference proceedings. We will also exclude studies without a description of how patients with hypertension were identified.

#### Interventions

Of interest in this review are interventions addressing hypertension prevention, control, or both, in a broad perspective, using pharmacological or nonpharmacological approaches. We will also include nonspecific or multifaceted behavioral, educational, policy, or other types of interventions given what exists in the literature, subject to the level of information available from clinic-based and community-based studies.

Regarding the first review question, the included studies will cover exposure to the risk factors and determinants of hypertension. We will identify the incidence and prevalence of hypertension and its morbidity, mortality, and burden in adults exposed to these risk factors in Cameroon. Concerning the second review question, the included studies will address the strategies, intervention, and programs that have been designed, implemented, and evaluated for the prevention and control of hypertension in various settings and among various adult population subgroups in Cameroon.

#### Comparators

Given the broad perspective for interventions of interest, several comparisons will be relevant to include. Some may be more likely to come from hospital-based studies and others from population-based studies. We will draw on recommendations of the World Hypertension League [34,35] and the *Global Action Plan on Prevention and Control of NCDs 2013-2020* [8] to distinguish adult hypertensive versus normotensive populations. We will define people with normotensive BP as the adult population who have a measured SBP less than 140 mm Hg and DBP less than 90 mm Hg in the absence of treatment with medication for high BP. We will define people with optimal BP as the adult population having measured SBP less than 120 mm Hg and measured DBP less than 80 mm Hg in the absence of treatment with medication for high BP.

With regard to the first review question, the comparison among adults within subgroups will be multilevel (type of place of residence [rural, urban, or both] nested within 5 agroecological zones nested within the 10 regions of Cameroon). For the second review question, the control adults will be those not receiving the treatment or the intervention, or not participating in the program.

#### Outcomes

The ability to reliably evaluate the impact of interventions and changes in hypertension prevention and control is critical if the burden of hypertension-related disease is to be reduced [35]. End points important for decision making of primary interest in this review are BP testing, diagnosis of hypertension, awareness of hypertension, treatment of hypertension, BP medication adherence, and BP control for hypertension. End points important for decision making that are secondary outcomes in this review are morbidity attributed to hypertension, mortality attributed to hypertension, and screening for modifiable risk factors in adults with hypertension (or with prehypertension). As some outcomes may be reported as a composite measure, we will extract all composite and individual outcomes as reported in the studies. Due to possible variation in definitions over time of hypertension and hypertension-related diseases and conditions, we will extract definitions of outcomes as reported in individual studies. We will extract outcomes in all data forms (eg, dichotomous, continuous) as reported in the included studies.

For the hypertension outcomes, inclusion criteria comprise hypertension indicators reported or deducible from subgroup estimates, at a population level in population-based surveys or in primary health care; hypertension assessed as BP 140/90 mm Hg or higher, or hypertension assessed as BP 160/95 mm Hg or higher, or other assessment criteria; use of antihypertensive drugs; or self-reported physician-diagnosed cases. We will use both self-reported data from population-based surveys and physician-reported data, because the accuracy of self-reported diagnosis of hypertension compared with chart reviews and physician-reported history has been demonstrated in various clinic and community settings [34,36-39]. In fact, the inclusion of self-reported diagnosis, including individuals who have controlled their BP using lifestyle changes as stand-alone therapy, increased the estimated prevalence of hypertension by 5% to 10% in the United States [40-42]. For meta-analysis, we will restrict analyses to studies for which hypertension was assessed as BP 140/90 mm Hg or higher, or use of antihypertensive drugs, or self-reported physician-diagnosed cases.

#### Study Design

We will include all clinic, hospital-, community-, and population-based studies, regardless of design. We will include studies conducted using quantitative, qualitative, or mixed research approaches. For quantitative studies, we will only include studies reporting the presence versus absence of at least one of the outcome variables in the total number of participants with or without a given exposure variable (2 × 2 table); such data will be sufficient to calculate odds ratios [43]. To maximize sensitivity, we will place no limitations on whether there was blinding for clinical information in a trial, whether there was a measure of intrarater or interrater reliability, or whether the level of experience or background (cardiologist, endocrinologist, neurologist, etc) of the raters is specified, although we will extract this information if available and it will contribute toward assessment of risk of bias.

#### Time Frame

We will select all journal articles and reports for inclusion if they were published up to December 31, 2016.

#### Setting

This review will focus on Cameroon. Situated in the crook of the Gulf of Guinea and with a surface area of 475,442 km^2^, the Republic of Cameroon is bounded to the northwest by Nigeria, to the north by Chad, to the east by the Central African Republic, and to the south by Congo, Equatorial Guinea, and Gabon [44,45]. It is commonly referred to as “Africa in miniature” because of its multitude of ecosystems and associated biodiversity, agroecological richness, cultures and traditions, and ethnolinguistic diversity, which together capture much of tropical Africa [44-49]. The country also harbors very ancient human populations with very intimate relationships with nature and where animals play important roles for the populations’ livelihood [48,49]. Globally, the incidence and pattern both of cardiovascular disease and cancer vary with the ecology of different regions [50-52]. Given the fragmented nature of most tropical ecosystems, the landscape epidemiological investigation of hypertension in Cameroon representing a variety of agroecosystem types with its 5 agroecological zones will offer a microcosmic portrait of hypertension in diverse ecosystems in tropical Africa; hence, what will be seen in miniature in Cameroon may be seen enlarged upon an SSA ecological scale. The 5 agroecological zones are highland and grassfield (West and Northwest regions), humid forest with monomodal rainfall (Littoral and Southwest regions), humid forest with bimodal rainfall (Centre, East, and South regions), high guinea savannah (Adamawa region), and Sudano-Sahelian (North and Far North regions) [53].

English and French are the 2 official languages spoken across its 10 regions (English is spoken by about 20% and French by about 80% of the population), with more than 250 ethnic groups speaking approximately 200 different dialects [44,45]. Cameroon’s major ethnic groups in terms of proportion are 38% Western Highlanders/Grassfielders (Bamileke, Bamoun); 12% Coastal Tropical Forest Peoples (Bassa, Douala, etc); 18% Southern Tropical Forest Peoples (Ewondo, Beti [Bulu and Fang subgroups], Maka, and Pygmies/Bakas); 14% Fulani (Islamic Northerners); and 18% Kirdi (non-Islamic Northerners).

The health system and health status vary widely by region and ecology, and several health care systems coexist. Cameroon’s public health sector is pyramidal in structure, with a centralized system of administration that runs from the central (ministry), through the intermediary (regional delegations), to the peripheral (health districts) levels. A total of 3 different levels of health care delivery services are evident: the tertiary, the secondary, and the primary services. Since 1992, the Minister of Public Health has promoted a national health policy of decentralization designed to maximize available resources at the district level; the nonprofit, private sector has an important place in Cameroon’s health system, offering a wide network of services throughout the country; the private, for-profit sector operates in the large cities, while traditional medicine is omnipresent. Traditional medicine—health practices, approaches, knowledge, and beliefs incorporating plant-, animal-, and mineral-based medicines, spiritual therapies, manual techniques, and exercises, applied singularly or in combination to treat, diagnose, and prevent illnesses or maintain well-being—is very popular in Cameroon [54]. This has prompted the government, in line with the provisions of the World Health Organization (WHO) strategies on traditional medicines [55], to take steps toward regulating the practice and use of traditional medicine in Cameroon. Notable steps taken as early as during the 1980s include the creation of the Traditional Medicine Service within the Unit of Community Medicine in the Yaoundé Central Hospital and the setting up of the Office of Traditional Medicine in the Ministry of Public Health [24]. Cameroon’s health care system is pluralistic in nature, characterized by multiple sources of financing and health care providers. The main financing sources are the government, public enterprises, foreign aid donors, private enterprises, households, religious missions, and nongovernmental organizations. Government health facilities, public enterprise health clinics, health facilities of religious missions and nongovernmental organizations, private clinics, pharmacies and drug retailers, and traditional doctors constitute the main providers [25]. Infectious diseases such as malaria, HIV/AIDS, and lower respiratory infections are still the primary causes of morbidity and mortality; however, in recent years, evidence has emerged that NCDs such as stroke, ischemic heart disease, diabetes, and cancers are also contributing causes [27].

#### Language

We will include all journal articles and reports in the English and French languages.

### Information Sources

We will develop literature search strategies using MeSH and text words related to the epidemiology and control of hypertension. We will search for relevant studies published in English or French from the following databases: PubMed, Medline, Embase, CINAHL, Web of Science, POPLINE, Scopus, and Banque de données en santé publique. We will use the appropriate Boolean operators to combine the following search terms: “hypertension,” “hypertensive,” “high blood pressure,” “raised blood pressure,” “screening,” “diagnosis,” “measurement,” “incidence,” “prevalence,” “awareness,” “morbidity,” “mortality,” “treatment,” “prevention,” “control,” and “Cameroon.” The titles and abstracts of the resulting studies will be screened for further review. We will also manually search for relevant gray literature from sources such as publications by the WHO, the African Development Bank, the World Bank, the Cameroon National Institute of Statistics, and the Cameroon Ministry of Public Health. We will include studies on hypertension (1) in human male and female adults aged 18 years or older (2) published up to December 31, 2016 that (3) are either clinic or community-based, (4) used qualitative, quantitative, or mixed research methods and (5) were published in English or French.

In regard to gray literature, we will search the proceedings of the European Society of Hypertension, the South African Hypertension Society, the American Society of Hypertension, the World Hypertension Congress, and the Annual General Meeting of the American Society of Hypertension. We will also search the World Heart Federation, International Society of Hypertension, World Hypertension League, American Heart Association, Cameroon Cardiac Society, Pan-African Society of Cardiology, *Integrated Blood Pressure Control* journal, the Hypertension, Diabetes and Dyslipidemia Conference, and Canadian Hypertension Congress-General through PapersFirst (WorldCat), ProceedingsFirst (WorldCat), and Web of Science. The websites of pertinent organizations will also be examined for articles and the names of researchers.

We will undertake several additional approaches to increase our retrieval of relevant articles and to ensure literature saturation. We will search our personal files to make sure that we have captured all relevant material. We will circulate a bibliography of the included articles to the systematic review team, as well as to hypertension experts identified by the team. To ensure that we have not missed studies, we will hand search hypertension-relevant journals such as *Hypertension*, *International Journal of Hypertension*, *Journal of the American Society of Hypertension*, *American Journal of Hypertension*, *Hypertension Research*, *Journal of Human Hypertension*, *The Journal of Clinical Hypertension*, *Current Hypertension Reports*, *American Heart Journal*, *International Journal of Cardiology*, *European Heart Journal*, and *Cardiovascular Journal of Africa*. These journals are considered to have the highest impact for the clinical subject of interest. Subject experts on hypertension in or on Cameroon will also be contacted to enquire about any studies felt to be applicable but not retrieved by our search strategy. Articles meeting the inclusion criteria will be searched in the Web of Science and Elsevier ScienceDirect for articles citing these articles. We also review references from included articles.

### Search Strategy

An expert librarian at the paramedical library of the University of Montreal provided assistance for the selection of relevant databases, keywords, and MeSH terms, and the construction of an effective combination of search terms involving breaking down the 2 review questions into concepts. We will seek qualitative, quantitative, and mixed-methods studies. The search for studies will be comprehensive as determined by the 2 review questions and thorough searching by using a variety of search methods, including electronic and manual searches, and by searching multiple and conceivably intersecting resources so as to minimize publication and language biases. We will also search more widely to identify research results circulated as reports or discussion papers, as well identifying gray literature; an expert librarian at the paramedical library of the University of Montreal will also provide access to collections of gray literature. We will acquire the full reports for all unpublished literature before considering whether to include their results in our systematic review. These various strategies for searching studies for the planned review will reduce the impact of publication bias.

We will use the bibliographic software EndNote X8 (Clarivate Analytics) to record and manage references, which will help in documenting the process, streamlining document management, and making the production of reference lists for reports and journal articles easier.

Table 1 lists the search conceptualization and draft search strategy as will be performed in the Ovid Medline database for the 2 review questions (search strategy). We will record and explain any changes or amendments we make. We will record all searches, including Internet searches, hand searching, and contact with experts.

**Table 1:** Ovid Medline search strategy (1946 to December 31, 2016).

Search no.	Query
1	exp Epidemiology/ OR exp Epidemiologic Methods/ OR exp Epidemiologic Studies/
2	Incidence/
3	Prevalence/
4	Awareness/
5	risk factors/
6	Protective Factors/
7	“Social Determinants of Health”/
8	exp Risk/
9	exp Morbidity/
10	exp Mortality/
11	Comorbidity/
12	1 OR 2 OR 3 OR 4 OR 5 OR 6 OR 7 OR 8 OR 9 OR 10 OR 11
13	(epidemiolog* OR incidence OR prevalence OR awareness OR “risk factor*” OR burden OR risk* OR determinant* OR morbidit* OR mortality OR comorbidit*).mp. [mp=title, abstract, original title, name of substance word, subject heading word, keyword heading word, protocol supplementary concept word, rare disease supplementary concept word, unique identifier]
14	12 OR 13
15	exp Hypertension/
16	(Hypertens* OR “raised blood pressure” OR “high blood pressure” OR “systolic blood pressure” OR “diastolic blood pressure”).mp. [mp=title, abstract, original title, name of substance word, subject heading word, keyword heading word, protocol supplementary concept word, rare disease supplementary concept word, unique identifier]
17	15 or 16
18	Cameroon/ NOT Nigeria.mp. [mp=title, abstract, original title, name of substance word, subject heading word, keyword heading word, protocol supplementary concept word, rare disease supplementary concept word, unique identifier]
19	Cameroun.mp. [mp=title, abstract, original title, name of substance word, subject heading word, keyword heading word, protocol supplementary concept word, rare disease supplementary concept word, unique identifier]
20	(“Western ADJ highland*”OR Grassfield* OR Bamileke OR Bamoum OR Bamoun OR “Coastal ADJ tropical ADJ forest” OR Bassa OR Douala OR “Southern ADJ tropical ADJ forest” OR Ewondo OR Beti OR Bulu OR Fang OR Maka OR Pygm* OR Baka OR Fulani OR Fulbe OR Peul OR Peuhl OR “Islamic ADJ Northerners” OR “Non-Islamic ADJ Northerners” OR “Centre ADJ region” OR “East ADJ region” OR “South ADJ region” OR “Littoral ADJ region” OR “South ADJ West ADJ region” OR “West ADJ region” OR “North ADJ West ADJ region” OR “Adamawa ADJ region” OR “North ADJ region” OR “Far-North ADJ region” OR “Centre ADJ province” OR “East ADJ province” OR “South ADJ province” OR “Littoral ADJ province” OR “South ADJ west ADJ province” OR “West ADJ province” OR “North ADJ West ADJ province” OR “Adamawa ADJ province” OR “North ADJ province” OR “Far-North ADJ province”).mp. [mp=title, abstract, original title, name of substance word, subject heading word, keyword heading word, protocol supplementary concept word, rare disease supplementary concept word, unique identifier]
21	18 OR 19 OR 20
22 (Q1^a^)	12 AND 17 AND 21
23	Mass Screening/
24	exp Diagnosis/
25	exp Therapeutics/
26	exp Tertiary Prevention/ OR exp Secondary Prevention/ OR exp Primary Prevention/
27	exp Disease Management/
28	Early Medical Intervention/
29	exp Policy/
30	23 OR 24 OR 25 OR 26 OR 27 OR 28 OR 29
31	(Control OR screening OR diagnosis OR measurement OR treatment OR prevention OR management OR intervention OR program OR policy OR action OR Trial).mp. [mp=title, abstract, original title, name of substance word, subject heading word, keyword heading word, protocol supplementary concept word, rare disease supplementary concept word, unique identifier]
32 (Q2^b^)	22 AND 31

^a^Q1: review question 1.

^b^Q2: review question 2.

### Updating Literature Searches

We will update the search toward the end of the review, if we carried out the initial searches some time before undertaking the final analysis (eg, 6 months). Updating literature searches will involve rerunning the searches to ensure that no recent articles are missed, after being validated to ensure that the search strategy retrieves a high proportion of eligible studies found through any means and information sources for the planned review. To do this successfully, we will record the date the original search was conducted and the years covered by the search. When doing update searches if necessary, we will use the update date field rather than the actual date, thereby ensuring that we will identify any additions to the database after we conducted the original search. For databases that do not include an update date field, we will run the whole search again and then use reference management software EndNote X8 to remove those records that we have already identified and assessed.

### Study Records

#### Selection Process

The selection process will be wholly computerized and the search results will be provided in electronic format, so that they can be imported into the reference management software, EndNote X8, being used by the review team. To determine eligibility of identified studies, parallel independent assessments will be conducted to minimize the risk of errors by two review authors, who will independently screen titles and abstracts during the study selection process, based on the inclusion and exclusion criteria. Neither of the review authors will be blinded to the journal titles or to the study authors or institutions. We will obtain full texts for all titles that appear to meet the predefined eligibility criteria or where there is any uncertainty. We will seek additional information from study authors where necessary to resolve questions about eligibility. Disagreements between the 2 reviewers will be resolved using consensus and arbitration as appropriate by a third member of the review team, or by contacting authors of original studies to resolve any uncertainties; interrater agreement will not be calculated. This should create a sensitive search strategy to capture relevant studies for this review. We will document the study selection process, including detailing reasons for excluding studies. These preventive steps will help minimize bias and errors in the study selection process.

##### Piloting the Study Selection Process

To minimize bias, we will pilot the selection process by applying the inclusion criteria to a sample of articles to check that they can be reliably interpreted and that they classify the studies appropriately. We will use the pilot phase to refine and clarify the inclusion criteria and ensure that the criteria can be applied consistently by more than one person. Piloting will also give an indication of the likely time needed for the full selection process.

##### Reporting Study Selection

To document the study selection process, we will produce a flow chart showing the number of studies or articles remaining at each stage. This flow chart will adhere to recommendations for reporting and presentation of a flow chart when reporting systematic reviews with or without a meta-analysis [33]. The subject headings of studies meeting the inclusion criteria will be examined to ensure that all relevant terms have been captured. If needed, additional searches will be undertaken. A list of studies excluded from the review will also be reported with the reasons for exclusion and will be included in the report of the review as an appendix. This list will be restricted to “near misses” (ie, those studies that only narrowly failed to meet inclusion criteria and that readers might have expected to see included) rather than all the research evidence identified.

#### Data Collection Process

We will keep a record of all searches and search decisions to ensure reproducibility. Search results will be exported to the citation management software EndNote X8. Duplicates will be removed and retained separately. The resulting references will be exported separately to the 2 reviewers for independent review using Excel version 2010 (Microsoft Corporation). As with screening, data extraction will be carried out in duplicate by 2 independent reviewers to reduce bias and reduce errors in data extraction [56]. Using this standardized data collection format, 2 review authors will extract data independently and in duplicate from each eligible study. To ensure consistency between the 2 reviewers, we will conduct calibration exercises before starting the review. Data abstracted will include study design, period of data collection, year of publication, study setting, geographic coverage, demographic information, intervention details, and all reported hypertension-related outcomes. Disagreements between the 2 reviewers will be resolved by a third member of the review team or by contacting authors of original studies to resolve any uncertainties.

Data extraction will also involve collection and scrutiny of detailed raw databases in collaboration with authors of the original study, some of whom are members of the review team (see Conflicts of Interest, below). We will confirm the accuracy of the extracted information to be included in the systematic review and meta-analysis with original researchers, by sending them a copy of the draft review when available with all working files used for data synthesis, assessment of risk of bias, and meta-analysis.

Since data in primary studies may not always be presented in a format that is useful to systematic reviewers, we will contact authors for missing information about exposure and outcome variables and ask authors for this information. We plan to contact authors of included studies by email and will endeavor to make up to a maximum of 3 email attempts to obtain missing information.

#### Dealing With Duplicates

We will look for duplicate publications of research results to ensure they are not treated as separate studies in the planned review. Multiple articles of a study may be published for reasons such as translation of results to different audiences or reporting of different outcomes, but this may be often concealed (ie, not cross-referenced to one another), and neither authorship nor sample size is a reliable criterion for identifying duplication [57]. Multiple reports from the same study may include identical samples with different outcomes reported or increasing samples with the same outcomes reported. Multiple reporting can lead to biased results, as studies with significant results are more likely to be published or presented more frequently, leading to an overestimation of treatment effects when findings are combined [58]. When we identify multiple reports of a study, we will treat them as a single study, and we will make reference to all the publications. We will also compare multiple publications for any discrepancies, which we will highlight, and contact the study authors for clarification.

#### Data Management

To ensure the efficient management of retrieved records, 1 review author will screen the references and record decisions about which documents to obtain and how to code these decisions. We will have a record of decisions made for each article. Decisions about rejecting or obtaining documents will not be made blind to others’ decisions, and documents received will be stored in our PRONUSTIC Research Laboratory at the University of Montreal. In addition, 1 review author will be responsible for identifying and removing duplicate references, ordering interlibrary loans, recording the receipt of documents, and following up on nonarrivals. The bibliographic software EndNote X8 will be used to record and manage references, in documenting the process, streamlining document management, and making the production of reference lists for reports and journal articles easier. We will create a “library” (database) of references, so that the whole review team can share information. One review author will be responsible for the library of references. These data management steps will contribute to minimize bias and errors in the data extraction process.

We will search each of the databases using the selected keywords, together with a set of MeSH terms. The retrieved search results will be exported to the reference management software EndNote X8, where duplicates will be excluded. The first step in the selection process will then be completed by 2 independent reviewers, reading through the titles and abstracts of the articles retained. Articles not meeting the eligibility criteria will be excluded. The remaining articles with available full text will be further reviewed by the 2 reviewers based on the defined exclusion criteria, with each reviewer independently reading each of them. Disagreements will be resolved by consulting a third reviewer and during reconciliation meetings. Full-text versions of selected studies will then be examined and coded by 2 reviewers working independently. They will seek to identify relevant information in line with the review questions and the aim of the review. Once all pertinent literature has been collected and assessed by the 2 reviewers, the main findings of the retained articles will be extracted and integrated into a synthesis table (data extraction form) to ease the manipulation of findings and to organize them for evidence synthesis. Coding will enable reviewers to obtain information on the design, period of data collection, year of publication, study setting, and geographic coverage (ie, rural, urban, or both). Data to be extracted from each article will include author name (s), date of data collection, date of publication, study design, age range of participants, type of place of residence of participants, and ecological zone and region of residence of participants within the country. Coded data will then be extracted using a data extraction sheet, which will be designed based on the objectives of the review. That datasheet will be shared among review team members and will facilitate collaboration among reviewers.

#### Data Items

This review will assess the epidemiological landscape of hypertension in terms of geographic coverage (ie, rural, urban, or both), ecological zones and regions of residence of study participants aged 18 years or older, and costs (at the individual, family, community, and national levels) on the one hand, and the control of hypertension on the other hand, both from clinic-based and community-based studies.

From each study, we will extract outcome variables for hypertension and hypertension-related diseases, conditions, and events; hypertensive patient characteristics (eg, average age, sex, age at diagnosis); study design; sample size; and type and source of financial support. Multimedia Appendix 1 presents further definitional details on primary and secondary outcomes to be included in the review. Variables for which data we will seek data are as follows. (1) The frequency and distribution of hypertension and its population impact as measured by its incidence, prevalence, morbidity, or mortality due to hypertension. (2) The occurrence and causes of health effects of hypertension in population subgroups in Cameroon. (3) The awareness of hypertension in the Cameroonian population. (4) The impact of individual-level risks and protective factors (background risk factors such as age, sex, level of education, and genetic composition; behavioral risk factors such as tobacco use, unhealthy diet, and physical inactivity; and intermediate risk factors such as elevated blood lipids, hypertension comorbidities, and overweight and obesity), family-level factors (eg, age of the head of household, family income, family size, family composition, and family socioeconomic status), and community-level factors (social and economic conditions such as poverty level, employment level, socioeconomic status, and social and health infrastructures; environmental factors such as climate or air pollution; and cultural factors such as practices, norms, and values; and urbanization, which influences housing, and access to products and services) in the Cameroon context on morbid conditions. (5) The strategies that have been designed and implemented for the prevention and control of hypertension in various settings and among various population subgroups in Cameroon.

When necessary, we will approximate means and measures of dispersion from figures in the reports. Whenever possible, we will use results from an intention-to-treat analysis. If we cannot calculate effect sizes, we will contact the study’s authors for additional data.

### Outcomes and Prioritization

We will seek the following primary outcomes: diagnosis, evaluation, detection, incidence, prevalence, awareness, treatment, treatment adherence, management, prevention, and control of hypertension.

We will seek the following secondary outcomes: hypertension-related morbidity and mortality, costs of treatment of hypertension, and influential risk factors of hypertension amenable to intervention and prevailing in Cameroon (eg, overweight and obesity) [25].

Specification of the duration of hypertension or a measure of the severity of hypertension by physical examination, retinal funduscopy, chest roentgenogram, electrocardiogram, blood urea nitrogen measurement, and urinalysis using a range of clinical assessments, exercise tests, biochemical markers, and echocardiographic and hemodynamic assessments (eg, presence of end-organ damage, including the kidneys, eyes, and heart) will not be required for inclusion, although this information will be extracted if available and will contribute toward assessment of risk of bias.

### Risk of Bias of Individual Studies

Our review will critically appraise relevant studies conducted using either quantitative, qualitative, or mixed research approaches. To ease the assessment of possible risk of bias (or “quality”) for each study and the evaluation of the overall strength of evidence of the planned review, we will use a tool designed and validated for this purpose by Downs and Black [59]. The Downs and Black checklist consists of 27 items that cover the following risk-of-bias domains: reporting, external validity, internal validity (bias and confounding), and power. The psychometric properties of the Downs and Black checklist have been validated previously [59]. For each domain in the tool, we will describe the procedures undertaken for each study. In the event of insufficient detail reported in the study to judge the risk of bias, we will contact the study investigators for more or missing information. We will judge the possible risk of bias on each of the 6 domains based on the extracted information.

Two review authors with extensive risk-of-bias assessment experience will independently perform these assessments. The 2 review authors will not be blinded to studies, agreement between them will not be evaluated, and disagreements between them will be resolved first by discussion and then by consulting a third author for arbitration. We will compute graphic representations of potential bias within and across studies using Excel version 2010. We will first consider each item in the risk-of-bias assessment independently, and then we will collate and assign an overall score. Scores range from 0 to 28, with higher scores indicating a better methodological quality of the study. The following cut offs have been suggested to categorize studies by quality: excellent (26-28), good (20-25), fair (15-19), or poor (≤14), based on how likely further research is to change the confidence in the estimate of the effect [60]. Risk-of-bias assessments will be incorporated into data synthesis through subgroup analyses and their potential influence on findings of the planned review.

### Data

Our planned systematic review aims to synthesize the results of all relevant studies on hypertension in Cameroon. Synthesis will involve the collation, combination, and summary of the findings of individual studies included in the systematic review. Synthesis will be done quantitatively using meta-analysis or, if formal pooling of results is inappropriate, through a narrative approach. As well as drawing results together, synthesis will consider the strength of evidence, explore whether any observed effects are consistent across studies, and investigate possible reasons for any inconsistencies. This will enable reliable conclusions to be drawn from the assembled body of evidence.

We will summarize and present our findings in text and tables to describe the characteristics of included studies, as well as their findings, in line with the objectives of the review and the 2 review questions. We will present results in order by (1) review question, (2) within each review question, in order of primary and then secondary outcomes, and (3) within each outcome, in order of “concept” in the title of the manuscript. To be comprehensive, we will include studies with any level of risk of bias in our analyses, and we will present information in tables by risk-of-bias level.

#### Quantitative Synthesis

We will use quantitative synthesis involving meta-analyses using a random-effects model and a metaregression model if the included studies are sufficiently homogeneous in terms of design and comparator. In Multimedia Appendix 1, we describe, with reference to the PICO (population, intervention or indicator, comparison, outcome) criteria, primary and secondary outcomes by study setting (clinic vs community or population-based studies) that we will consider for such statistical synthesis. These outcomes will rely on measured SBP and DBP, self-reported diagnosis of hypertension by a health professional, and self-reported current use of antihypertensive medication.

For analyses of dichotomous-event data (eg, hypertension-related morbidity and mortality; hypertension with other comorbidities present), we will use risk ratio with 95% CI. Continuous outcomes will be analyzed using weighted mean differences (with 95% CI) or standardized mean differences (95% CI) if different measurement scales are used. Skewed data and nonquantitative data will be presented descriptively.

#### Unit of Analysis

The unit of analysis will be the individual study.

#### Dealing With Missing Data

When data are missing, we will attempt to contact the original authors of the study to obtain the relevant missing data. Important numerical data will be carefully evaluated. If we cannot obtain missing data, we will use an imputation method. We will use sensitivity analysis to assess the impact on the overall treatment effects of inclusion of trials that do not report an intention-to-treat analysis, have high rates of participant attrition, or have other missing data.

#### Assessment of Heterogeneity

We will test clinic and community heterogeneity by considering the variability in participant factors across studies (eg, age, sex, type of place of residence, ecological zone of residence, hypertension with or without comorbidity) and study factors (eg, study location, study design, year of data collection, type of BP measurement device used). Statistical heterogeneity will be tested using the chi-square test (significance level: 0.1) and I^2^statistic (0% to 40%: might not be important; 30% to 60%: may represent moderate heterogeneity; 50% to 90%: may represent substantial heterogeneity; 75% to 100%: considerable heterogeneity) [61]. If heterogeneity among the studies is high (I^2^≥50% or *P*<.1), we will analyze the study design and characteristics in the included studies. We will try to explain the source of heterogeneity by subgroup analysis or sensitivity analysis. The pooled odds ratio (or rate ratio) will be calculated and between-study heterogeneity will be determined by visual inspection of the forest plots and with consideration of the I^2^statistic. The Egger test and visual inspection of funnel plots will assess small study effects [62].

#### Statistical Data Synthesis

Meta-analysis will be carried out on the published results of relevant empirical studies on hypertension in Cameroon to address the 2 review questions. Pooled analyses will be undertaken for relevant studies of the adult population aged 18 years or older, for each review question and for each outcome of interest in turn. Each outcome will be combined and calculated using the statistical software STATA 13.1 (StataCorp LP). If we observe statistical heterogeneity (ie, I^2^≥50% or *P*<.1), we will choose the random-effects model because this model assumes that the aggregated results should be valid beyond the sample used by each study and are based on both participant characteristics and study design [62]. If heterogeneity is considerable (ie, I^2^≥75%), we will not perform a meta-analysis; instead, we will do a narrative, qualitative summary.

#### Qualitative or Narrative Synthesis

Due to diversity in study populations, interventions, and outcomes, quantitative synthesis may not be appropriate. If this happens, we will provide a systematic narrative synthesis including information presented as text and in tables to summarize and explain the characteristics and findings of the included studies. The narrative synthesis will explore the relationship and findings both within and between the included studies, in line with the guidance from the Centre for Reviews and Dissemination [63].

The strategy for data narrative synthesis will be as follows. We will present the general characteristics of the reviewed studies in tables. Content analysis will be carried out by each of the 2 independent reviewers of each study to identify therein evidence on the epidemiology and control of hypertension in various settings and among various population subgroups in Cameroon.

#### Analysis of Subgroups or Subsets and Metabiases

For each review question, we plan to carry out pooled analyses for examining the extent to which measured indicators of the epidemiology and control of hypertension vary by type of place of residence (ie, rural, urban, or both) and agroecological zone or region in Cameroon, if data are appropriate for such analyses. Separate meta-analyses will be done for studies conducted in people with hypertension only versus hypertension with other conditions or diseases.

We will incorporate the risk-of-bias assessments into data synthesis through pooled analyses undertaken for exploring how a potential source of bias may influence our review findings, by presenting estimates of prevalence, awareness, and risk factors of hypertension by quality category (ie, excellent, good, fair, or poor).

We will explore the robustness of meta-analyses by using pooled analyses undertaken for investigating possible causes of between-study heterogeneity and variability based on (1) the characteristics of participants (eg, age, sex, type of place of residence, agroecological zone or region of residence), and (2) the characteristics of studies (eg, sample size, study location, study design, year or period of data collection).

Sensitivity analysis will be performed to explore the source of heterogeneity by doing subgroup or metaregression analyses according to risk of bias by comparing the high-risk versus the low-risk studies. Pooled sensitivity analyses will be performed with a hypertension definition and measurement procedure (duration of rest prior to measurement, measurement arm, position of patient during measurement, BP measuring device, cuff size, number of measurements taken, and interval between measurements).

### Dissemination

To ensure that the essential messages from the planned review reach the appropriate audiences, we will disseminate the review findings (1) in Cameroon, especially to relevant ministries, institutions, and organizations involved in hypertension and comorbidities; (2) through national and international conferences; and (3) through publication in peer-reviewed journals.

### Ethical Approval of the Protocol

Since this will be a systematic review of published literature, we required no ethical approval for developing this protocol. However, we will ensure that all studies included in our review provided evidence of ethical approval and informed consent from all patients or respondents where required.

Data from studies included in the review will have been obtained with participant consent and ethical approval. If this is not stated in the article, we will contact the authors for confirmation.

Our funding bodies or the institutions of affiliation did not require that they formally approve the protocol, and they took no part in its design or development.

### Protocol Amendments During the Review

To ensure that our review is useful to end users, we will readily amend the protocol during the review if required, should consideration of the primary research raise questions that we did not anticipate at the protocol stage. If such consideration results from a clearer understanding of the review question(s), we will carry out documented and justified amendments to the protocol. In that case, in the report of the review findings, we will distinguish between the initial review question(s) and any subsequent amendments. Protocol amendments (if any) will be documented in a protocol addendum and in the final report of the review.

## Results

This review is part of an ongoing research program on disease prevention and control in the context of the dual burden of communicable diseases and NCDs in Africa. The first results are expected in 2017.

## Discussion

Studies of hypertension detection, prevalence, awareness, treatment, management, and control over the last 70 years or so show globally increasing hypertension disparities across geographic and ecological settings in a wide range of populations and health care systems of HICs and LMICs [12,51,64-67], as well as strikingly divergent portraits across countries. The highest BP levels have shifted from HICs to low-income countries in South Asia and SSA, while BP levels have been consistently high in Central and Eastern Europe [12,51,67]. The global prevalence of raised BP in adults aged 18 years or older was 22% in 2014, with Africa being the most affected region with a 30% prevalence [3]. By and large, published international and WHO guidelines are unsuitable for application in many ethnically diverse populations of countries in SSA because their economic, ecological, sociocultural, and health care environments, poor infrastructure hindering health-promoting lifestyles, and unaffordability for the patients impede the adoption of recommended nonpharmacological and pharmacological treatment [23,30,32,68-70]. Moreover, good-quality data on hypertension in different ethnic populations in much of SSA are notoriously missing in the treatment guidelines, which are largely based on white populations, whereas cardiovascular disease varies with ethnic origin [71]; of the more than 180 studies that met the standards of the Eighth Joint National Committee, fewer than 30 were from nonwhite, non-African American groups [9]. National hypertension evidence in SSA is urgently needed for translating new knowledge and providing up-to-date information and recommendations for hypertension prevention and control to health care providers at different levels of the health care system pyramid, including primary health care and community programs at the base of the pyramid for the primary prevention of hypertension especially.

The world population is projected to reach 9.7 billion by 2050 and, with the highest rate of population growth, Africa is expected to account for more than half of the world’s population growth between 2015 and 2050 [72]. Hypertension in SSA is viewed as “a massive and increasing health disaster” [73]. It is estimated that the number of adults with raised BP increased from 594 million in 1975 to 1.13 billion in 2015, with the increase largely in LMICs; moreover, the global increase in the number of adults with raised BP is a net effect of increase due to population growth and aging on the one hand, and of decrease due to declining age-specific prevalence of hypertension on the other [11,12]. Such divergent trends are indicative of underlying large and widening disparities in hypertension prevention and control across countries [51,65-67]. They are also expressions of fundamental within-country differences between rural and urban communities in hypertension awareness, diagnosis, treatment, and management [19,23,51], and effective use and appropriateness of medications for controlling hypertension for the SSA populations. Current guidelines are generally extrapolations of findings among people of African descent living in Western countries [23,32], despite differences in prevailing influential country-specific factors affecting hypertension risk, including ecological factors [74-82], community endowments [83,84], and social disparities [85], above and beyond social and economic inequalities [86]. Indeed, advances in a wide range of biomedical, biological, behavioral, and social sciences have been expanding our understanding of how early and continuing environmental influences (the ecology) and genetic predispositions (the biologic program) independently or synergistically affect human behaviors, functions, and lifelong health [79,83,87-89]. It is estimated that incidence rates of hypertension range between 3% and 18%, depending on the age, sex, ethnicity, and body size of the population studied [90]. According to recent estimates, the geographical variation in the distribution of hypertension is intensifying in several regions of the world, notably in LMICs, which now simultaneously have to grapple with the double burden of communicable diseases and NCDs despite limited health care resources [91-93].

These divergent trends and widening disparities call for urgent interdisciplinary and intersectoral collaborative endeavors between national and international stakeholders and researchers to combat the rising hypertension burden in LMICs. This could be done using country-specific knowledge bases and circumstances to develop or improve national hypertension guidelines for the control and prevention of hypertension, in order to reverse these trends and reduce the hypertension-related preventable and avoidable burden of morbidity, disability, and mortality in LMICs.

What is urgently needed is a better and locally situated understanding of the risk and protective factors associated with within-country disparities in hypertension prevention and control in the context of these divergent trends across countries. While we are learning more about levels and trends across countries from models providing estimates using existing and often defective but comparable data [11,12], there is an alarming lack of local data. Such data are more informative for designing and monitoring the effectiveness of the implementation of nationally appropriate policies, standards, and guidelines for medical practice in primary care typically found in the health care environments of LMICs in general and those of SSA in particular.

The evidence assembled from the planned review will fill some of the gaps in knowledge in these areas for Cameroon. With limited human and financial resources and increasing rates of NCDs, paramount among which is hypertension, African countries such as Cameroon must prioritize more cost-effective preventive approaches tailored to local circumstances and that can reach not only hypertensive patients coming to health facilities, but also the overwhelming majority of the population, who rarely attend health care facilities and yet are at risk of hypertension-related diseases, conditions, and events. Well-informed and trained stakeholders through the health system pyramid urgently need such evidence for fulfilling their important role in disease prevention (primary, secondary) and control, as they provide a primary point of contact for individuals who are healthy, at risk, or have diagnosed chronic illnesses such as hypertension. These stakeholders are also trusted sources of health information. Indeed, the World Health Assembly endorsed the WHO Global Action Plan for the Prevention and Control of Noncommunicable Diseases 2013-2020 in May 2013, according to which governments from member states were urged to (1) set national NCD targets for 2025 based on national circumstances, and (2) develop multisectoral national NCD plans to reduce exposure to risk factors and enable health systems to respond in order to reach these national targets in 2025 [8]. For raised BP, the target is a 25% relative reduction in the prevalence of raised BP or to contain the prevalence of raised BP, according to national circumstances; the indicators are the age-standardized prevalence of raised BP among persons aged 18 years or older (defined as SBP ≥140 mm Hg, DBP ≥90 mm Hg, or both) and mean SBP. The Global Plan provides a roadmap for country-led action for the prevention and control of NCDs; any effective action by a national government will depend on the extent of understanding of its country-specific circumstances, for which there are huge data needs, and gaps in knowledge and evidence, for African countries [23,30,32,67-70,91]. There is an urgent need to systematically map evidence on hypertension and comorbidities from clinic-based and community-based data reflecting within-country circumstances, so that the implementation of strategies and actions to curb the epidemic of hypertension is evidence based at the local or hospital level, where the costs, impacts, and benefits of health technologies and strategies can be directly assessed. Such assessment will have an impact on a hospital’s budget, clinical practices, and patient outcomes. It is also needed to better inform health care costs, which have become a primary concern of public policy in HICs, and increasingly in LMICs, due to the growing size of the aging population. The prevention and control of NCDs hinges on hypertension, which is the most common noncommunicable condition seen in primary care in HICs and LMICs alike [9,11,64,66,94].

The epidemiology and control of hypertension within the national circumstances of LMICs, which are increasingly bearing the greatest burden of NCDs in general and hypertension in particular, have only started to be fully described and appreciated in the continuing epidemiological landscape of the dual burden of communicable diseases and NCDs in SSA [88]. Much research still remains to be done into how the risk factors for hypertension are distributed in subpopulations across the ecological and areal contexts of these countries, especially across the sociocultural and environmental diversity of a country such as Cameroon, where patients with hypertension-related diseases, conditions, and events are a large portion of adults with cardiovascular diseases [27].

This review will shed light on the epidemiological landscape of hypertension in Cameroon, as well as the control strategies that have been designed and successfully implemented to mitigate the burden of hypertension. It will depict the quality and quantity of hypertension-related research output in Cameroon. This information will be relevant for mapping out mitigation strategies. We also hope that this review will highlight important aspects of hypertension in Cameroon that require further research and evidence. Cameroon is commonly denoted as “Africa in miniature,” and what will be documented in miniature in Cameroon may be enlarged to an SSA ecological scale for hypertension prevention and control.

## Appendix

Multimedia Appendix 1 Outcomes of hypertension, definitions, and core indicators for clinic-based or community-based studies.

## Abbreviations

BP: blood pressure
DBP: diastolic blood pressure
HICs: high-income countries
HIV: human immunodeficiency virus
LMICs: low- and middle-income countries
MeSH: Medical Subject Headings
NCD: noncommunicable disease
SBP: systolic blood pressure
SSA: sub-Saharan Africa
UI: uncertainty interval
WHO: World Health Organization
