# Supplementary material for: Diagnosis, Prevalence, Awareness, Treatment, Prevention, and Control of Hypertension in Cameroon: Protocol for a Systematic Review and Meta-Analysis of Clinic-Based and Community-Based Studies
Source: JMIR Res Protoc. 2017 May 29;6(5):e102. doi: 10.2196/resprot.7807 (PMC5468543; doi:10.2196/resprot.7807)
Supplement: Multimedia Appendix 1 [file resprot_v6i5e102_app1.pdf]

Outcomes of hypertension, definitions, and core indicators for clinic-based or community-based studies.

Core indicators at a population level in population-based surveys[a]				Core indicators in primary health care (PHC)		
Primary outcomes						
	Core indicators	Numerator	Denominator	Core indicators	Numerator	Denominator
Blood pressure testing				% of PHC patients, 18 years and over, who had their blood pressure measured in the past 24 months.	Number of PHC patients who had their blood pressure measured by their PHC provider in the past 24 months	Total number of PHC patients 18 years and over, in the past 24 months
Diagnosis of hypertension	Mean SBP	Sum of valid average SBP	Total number of respondents aged 18+ years who had a valid BP reading			
	Mean DBP	Sum of valid average DBP	Total number of respondents aged 18+ years who had a valid BP reading			
	Prevalence of hypertension	Respondents who have SBP ≥140 mm Hg or DBP ≥90 mm Hg or who report currently taking antihypertensive medication (definition A)	Respondents aged 18+ years	Prevalence of diagnosed hypertension	Patients who have SBP ≥140 mm Hg or DBP ≥90 mm Hg or who report currently taking medication for the treatment of HBP	Adult patients aged 18+ years
		Respondents who	Respondents			

		have SBP ≥140 mm Hg or DBP ≥90 mm Hg or who report currently taking medication for the treatment of HBP or who report having been diagnosed with hypertension by a health professional (definition B)	aged 18+ years			
	Prevalence of hypertension			Ratio of prevalence of diagnosed hypertension to the expected prevalence of hypertension	Prevalence of diagnosed hypertension	Expected age-adjusted prevalence of hypertension in the population[b]
Awareness of hypertension	Prevalence of awareness of hypertension	Respondents who report either having been diagnosed with HBP or who report being currently on antihypertensive medication	Respondents with hypertension according to definition A			
Management/ Treatment of hypertension	Prevalence of drug-treated hypertension	Respondents who report being currently on antihypertensive medication	Respondents with hypertension according to definition A	Appropriate treatment of patients	Registrants who are being treated with medications that do not include either a diuretic or calcium channel–blocking antihypertensive medication	PHC patients with hypertension

Management/ Medication adherence				Use of recommended antihypertensive drugs[c]	Registrants prescribed “core-recommended” antihypertensive drugs	PHC patients with hypertension
BP control for hypertension	Prevalence of drug-treated and -controlled hypertension	Respondents who report being currently on antihypertensive medication and have SBP <140 mm Hg and DBP <90 mm Hg	Respondents with hypertension according to definition A			
BP control for hypertension	Prevalence of controlled hypertension	Respondents who report being currently treated with medication for HBP or have been diagnosed with hypertension and have SBP <140 mm Hg and DBP <90 mm Hg	Respondents with hypertension according to definition B	Prevalence of controlled hypertension	Respondents who report being currently treated with medication for HBP and have SBP <140 mm Hg and DBP <90 mm Hg	PHC patients with hypertension aged 18+ years
BP control for hypertension				Uncontrolled hypertension 1	Registrants with SBP ≥140 mm Hg or DBP ≥90 mm Hg and cardiovascular disease, renal disease, or diabetes mellitus	Registrants with hypertension
BP control for hypertension				Uncontrolled hypertension 2	Number of PHC patients with SBP ≥160 mm Hg or DBP ≥100 mm Hg and not taking antihypertensive	Number of PHC patients with hypertension

					medication	
BP control for hypertension				Uncontrolled hypertension 3	Number of PHC patients with SBP ≥160 mm Hg or DBP ≥100 mm Hg taking antihypertensive medication	Number of PHC patients with hypertension
BP control for hypertension				“Resistant” hypertension[d]	Number of PHC patients with BP ≥160/100 mm Hg treated with three or more antihypertensive drugs	Adult patients with hypertension
BP control for hypertension				% of PHC patients, 18 years and over, with hypertension for duration of at least one year, who have blood pressure measurement control (<140/90 mmHg)	Number of PHC patients with hypertension for duration of at least one year, who have blood pressure measurement control (ie, <140/90 mmHg)	Total number of PHC patients, 18 years and over, with hypertension for duration of at least one year
Secondary outcomes						
Morbidity attributed to hypertension complications	Age-adjusted prevalence of morbidity from stroke and coronary heart disease	Respondents who report being currently treated with medication for HBP and who report having been	Respondents with hypertension according to definition A	Age-adjusted prevalence of morbidity from stroke and coronary heart disease	Patients with hypertension-related morbidity from stroke and coronary heart disease	Total number of PHC patients with hypertension

		diagnosed with stroke and coronary heart disease				
Mortality attributed to hypertension complications	Age-adjusted death rates from stroke and coronary heart disease	Deaths from stroke and coronary heart disease	Respondents with hypertension according to definition A, who report having been diagnosed with stroke and coronary heart disease	Age-adjusted death rates from stroke and coronary heart disease	Patients with hypertension-related deaths from stroke and coronary heart disease	Total number of PHC patients with hypertension
Screening for modifiable risk factors in adults with hypertension[e] (or with prehypertension)				% of PHC patients, 18 years and over, with hypertension (or with prehypertension) who received annual testing, within the past 12 months, for all (or any) of the following: fasting blood sugar, full fasting lipid profile screening, test to detect renal dysfunction (eg serum creatinine), blood pressure measurement, and	Number of PHC patients with hypertension who received annual testing, for all (or any) of the following: fasting blood sugar, full fasting lipid profile screening, test to detect renal dysfunction (eg, serum creatinine), blood pressure measurement, and obesity/overweight screening	Total number of PHC patients with hypertension (or with prehypertension) within the past 12 months

				obesity/overweight screening		

Notes: BP = blood pressure; HBP = high blood pressure; SBP = systolic blood pressure; DBP = diastolic blood pressure. Standard deviations of mean systolic and diastolic blood pressure and 95% confidence intervals for the proportions should be calculated. Each of these core indicators can be reported overall and by age group (eg, 18-29, 30-49, and 50-69 years) and sex, with crude and age-standardized (to the World Health Organization World standard) changes tracked over time. Reporting core indicators among people with diabetes, those with isolated systolic hypertension, and among sociodemographic groups is recommended as expanded analyses.

[a]In the STEPwise approach to Surveillance system, average systolic and diastolic blood pressure are calculated from two of three blood pressure readings, taken 3 minutes apart, with preference given to the last two measurements (World Health Organization, 2005).

[b]The observed prevalence of hypertension will need to be restricted to the same age range as the population survey for this indicator as hypertension prevalence rises with age.

[c]This performance indicator requires that the clinic has established a core set of medications. The core medications are selected to facilitate a standard approach to hypertension treatment with cost-effective and appropriate antihypertensive drugs.

[d]This definition of resistant hypertension is modified for a primary care low-resource setting.

[e]Definitions of Terms (CIHI, 2006). A PHC client/patient is an individual who has had contact with the provider at least once in the past year and has a record with the provider dating back at least two years. Full fasting lipid profile screening is a group of blood tests that are performed after fasting 14 hours and used to guide PHC providers in deciding how a person at risk should be treated. Lipid

profile includes total cholesterol, HDL cholesterol, LDL cholesterol and triglycerides. Report may also include HDL/cholesterol ratio or a risk score based on lipid profile results, age, sex, and other risk factors. Hemoglobin A1c test (also called the HbA1c or A1c test, or glycated/glycosylated hemoglobin) is a laboratory test that reflects the average glucose level over a 2- to 3-month period. Obesity/overweight screening measures may include the following: body mass index (BMI), a method of assessing body weight while taking height into account and calculated by dividing weight by height squared; waist to hip ratio (WHR)—BMI provides an index for obesity, but has limitations in predicting risk for cardiovascular events—enables prediction of cardiovascular risk. Creatinine is a substance in the blood to determine if the kidneys are functioning normally and to monitor treatment for kidney disease.

Sources: Adapted from Gee et al (2014), Campbell et al (2017), and CIHI (2006).

World Health Organization. WHO STEPS Surveillance Manual: The WHO STEPwise Approach to Chronic Disease Risk Factor Surveillance. Geneva, Switzerland: WHO Press, World Health Organization; 2005.

Gee ME, Campbell N, Sarrafzadegan N, et al. Standards for the uniform reporting of hypertension in adults using population survey data: recommendations from the World Hypertension League Expert Committee. J Clin Hypertens (Greenwich) 2014;16(11):773-81.

Campbell N, Ordunez P, Jaffe MG, et al. Implementing standardized performance indicators to improve hypertension control at both the population and healthcare organization levels. J Clin Hypertens (Greenwich) 2017;19(5):456-61.

Canadian Institute for Health Information. Health Care in Canada. Ottawa, ON: CIHI; 2006.
